# Abundance of gap junctions at glutamatergic mixed synapses in adult Mosquitofish spinal cord neurons

**DOI:** 10.3389/fncir.2014.00066

**Published:** 2014-06-26

**Authors:** Jose L. Serrano-Velez, Melanie Rodriguez-Alvarado, Irma I. Torres-Vazquez, Scott E. Fraser, Thomas Yasumura, Kimberly G. Vanderpool, John E. Rash, Eduardo Rosa-Molinar

**Affiliations:** ^1^Biological Imaging Group, University of Puerto RicoSan Juan, PR, USA; ^2^Molecular and Computational Biology Section, University of Southern CaliforniaLos Angeles, CA, USA; ^3^Department of Biomedical Sciences, Colorado State UniversityFort Collins, CO, USA; ^4^Program in Molecular, Cellular and Integrative Neurosciences, Colorado State UniversityFort Collins, CO, USA; ^5^Institute of Neurobiology, School of Medicine, University of Puerto RicoSan Juan, PR, USA

**Keywords:** connexin 35/36, connexins, dye-coupling, freeze-fracture replica immunogold labeling, gap junctions, mixed synapses, neurons, spinal cord

## Abstract

“Dye-coupling”, whole-mount immunohistochemistry for gap junction channel protein connexin 35 (Cx35), and freeze-fracture replica immunogold labeling (FRIL) reveal an abundance of electrical synapses/gap junctions at glutamatergic mixed synapses in the 14th spinal segment that innervates the adult male gonopodium of Western Mosquitofish, *Gambusia affinis* (Mosquitofish). To study gap junctions’ role in fast motor behavior, we used a minimally-invasive neural-tract-tracing technique to introduce gap junction-permeant or -impermeant dyes into deep muscles controlling the gonopodium of the adult male Mosquitofish, a teleost fish that rapidly transfers (complete in <20 mS) spermatozeugmata into the female reproductive tract. Dye-coupling in the 14th spinal segment controlling the gonopodium reveals coupling between motor neurons and a commissural primary ascending interneuron (CoPA IN) and shows that the 14th segment has an extensive and elaborate dendritic arbor and more gap junctions than do other segments. Whole-mount immunohistochemistry for Cx35 results confirm dye-coupling and show it occurs via gap junctions. Finally, FRIL shows that gap junctions are at mixed synapses and reveals that >50 of the 62 gap junctions at mixed synapses are in the 14th spinal segment. Our results support and extend studies showing gap junctions at mixed synapses in spinal cord segments involved in control of genital reflexes in rodents, and they suggest a link between mixed synapses and fast motor behavior. The findings provide a basis for studies of specific roles of spinal neurons in the generation/regulation of sex-specific behavior and for studies of gap junctions’ role in regulating fast motor behavior. Finally, the CoPA IN provides a novel candidate neuron for future studies of gap junctions and neural control of fast motor behaviors.

## Introduction

Electrical synapses, hereafter, gap junctions, are abundant, are evidenced by widespread coupling at birth, and until the central nervous system (CNS) matures, play a role in shaping and patterning neuronal connectivity in the invertebrate^**[1]**^ and vertebrate^**[2]**^ CNS (^**[1]**^Furshpan and Potter, 1959; Bennett et al., 1963; Robertson et al., 1963; Furshpan, 1964; Sotelo and Korn, 1978; Fulton et al., 1980; Vaney, 1991; Walton and Navarrete, 1991; Dermietzel and Spray, 1993; Peinado et al., 1993a,b; Kalb, 1994; Kandler and Katz, 1995; Wolszon, 1995; Laird, 1996; Rash et al., 1996; Bennett, 1997, 2000; Dermietzel, 1998; Edwards et al., 1999; Personius et al., 2001; Cohen-Cory, 2002; Herberholz et al., 2002; Mentis et al., 2002; Lewis and Eisen, 2003; Pereda et al., 2003; Scheiffele, 2003; Bennett and Zukin, 2004; Connors and Long, 2004; Montoro and Yuste, 2004; Szabo et al., 2004; Fan et al., 2005; Marin-Burgin et al., 2005, 2006, 2008; Phelan, 2005; Waites et al., 2005, ^**[2]**^Arumugam et al., 2005; Kamasawa et al., 2006; Chen et al., 2007; Chuang et al., 2007; McAllister, 2007; Norman and Maricq, 2007; Szabo and Zoran, 2007; Todd et al., 2010; Whelan, 2010; Hoge et al., 2011; Park et al., 2011; Hamzei-Sichani et al., 2012; Lynn et al., 2012; Sugimoto et al., 2013; Bautista and Nagy, 2014). As the CNS matures, gap junction uncoupling occurs as the initial electrical synapses switch to chemical synapses and the former concomitantly decrease in number in the adult.

Axo-axonal or dendrodendritic gap junctions are probably best known for coupling parvalbumin-containing γ-aminobutyric acid (GABA)ergic interneurons (Ins)to facilitate the synchronization of rhythmic oscillatory activity for neuronal communication in the adult neocortex (Galarreta and Hestrin, 1999, 2001a,b; Gibson et al., 1999; Fukuda and Kosaka, 2000, 2003; Bartos et al., 2002; Muller et al., 2005, 2006). Gap junction coupling has been identified in numerous neural microcircuits and has been linked to motor behavior; however, the extent to which gap junctions are linked to fast motor behavior has not been fully explored (Saint-Amant and Drapeau, 2000, 2001; Tresch and Kiehn, 2000; Bonnot et al., 2002; Drapeau et al., 2002; Kiehn and Tresch, 2002; Hervé et al., 2004; Rash et al., 2004, 2013; Söhl et al., 2005; Goodenough and Paul, 2009; Vervaeke et al., 2010; Pereda et al., 2013).

To assist in understanding gap junctions’ role in fast motor behavior, we used a minimally-invasive neural-tract-tracing/labeling technique to introduce either gap junction-permeant or -impermeant dyes into deep muscles controlling the gonopodium (a sexually dimorphic sperm transferring organ) of a “reference species”, the adult male Western Mosquitofish, *Gambusia affinis* (Mosquitofish) a small, sexually dimorphic teleost fish whose radical remodeling and shifting of the axial and appendicular musculoskeletal support facilitates an extremely rapid movement of the gonopodium to transfer encapsulated sperm bundles, spermatozeugmata, into the adult female reproductive tract (Rosa-Molinar et al., 1994, 1996, 1998; Rosa-Molinar, 2005; Rivera-Rivera et al., 2010).

**Figure 1:**
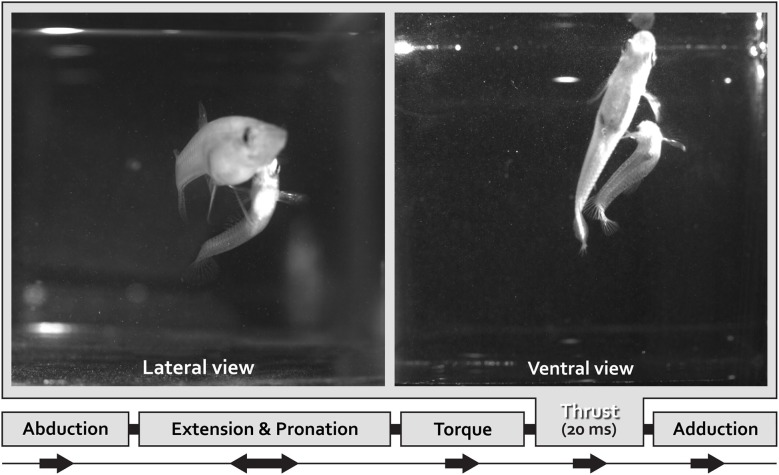
**One lateral and one ventral view of coital behavioral sequences filmed using high speed video at 500•frames•s–1.frames for a male Mosquitofish show the rapid movement portion of the circumduction of the gonopodium**. With the gonopodium abducted, the male approaches the female from behind and directly underneath her and then adducts the gonopodium. Just prior to circumducting, the gonopodium is extended and pronated to a point that is nearly parallel with the body. To transfer spermatozeugmata, the male Mosquitofish bends his body into an S-shape-type fast-start-like movement (see lateral and ventral view) during the torque-thrust motion of the circumduction of the gonopodium. The sequence ends with the gonopodium adducted. We analyzed the speed of the circumduction of the gonopodium (the specific movements were: abduction, extension and pronation, torque, thrust, and adduction), specifically the fastest portion of the sequence, the “thrust” movement (20 ms).

To transfer spermatozeugmata, the male Mosquitofish body bends into an “S-shaped fast-start” curvature defined as “torque” (Figure 1); simultaneously the gonopodium makes an extremely rapid directional movement defined as “thrust” (Figure 1; Weihs, 1973; Webb, 1976; Harper and Blake, 1990, 1991; Johnston et al., 1995; Rosa-Molinar et al., 1996; Domenici and Blake, 1997; Spierts and Leeuwen, 1999; Hale, 2002; Rosa-Molinar, 2005; Rivera-Rivera et al., 2010). The speed of the “torque/thrust” maneuver (complete in <20 mS), particularly of the “thrust” component, suggests that electrical and not chemical synapses are involved in controlling the finer aspects of Mosquitofish rapid motor behavior.

A simple dye-coupling assay combined with spinning disk confocal microscopy shows spinal motor neurons are dye-coupled to interneurons and reveals their unique arborization patterns and morphologies. Whole-mount immunohistochemistry combined with spinning disk confocal microscopy shows the dye-coupling to be via Cx35/36 puncta (i.e., gap junctions). Freeze-facture replica immunogold labeling (FRIL) confirms the immunohistochemistry results and reveals that the Cx35/36 puncta are, in fact, gap junctions at mixed synapses. Our results demonstrate the occurrence and abundance of axo-dendritic gap junctions at glutamatergic synapses between dye-coupled spinal motor neurons and interneurons in the adult Mosquitofish, particularly in a spinal region controlling an innate fast coital behavior of the adult male. The fundamental data and insights reported in this paper provide a basis for on-going work to unambiguously determine the connexin composition in apposed axo-dendritic gap junctions’ hemiplaques at glutamatergic synapses and to differentially map connexin distribution within the arbors of dye-coupled spinal motor neurons and interneurons in the adult Mosquitofish. The results also move us closer to attaining a clear understanding of the fundamental role of gap junctions in sculpting complex arborization patterns, morphologies, and synaptic connectivity of neurons during development and maturation of the CNS.

## Materials and methods

Eighty wild-type adult (female *n* = 40; male *n* = 40) Western Mosquitofish, *Gambusia affinis* (hereafter Mosquitofish) were used. All experimental procedures and care were approved and conducted according to Principles of Laboratory Animal Care (NIH publication No. 86–23, Rev. 1985 (Rosario-Ortiz et al., 2008)) and the University of Puerto Rico-Rio Piedras Institutional Animal Care and Use Committee guidelines. All fish were collected and maintained under permits issued by the Puerto Rico Department of Natural Resources.

### Dye-coupling assay

The 80 adult Mosquitofish were anesthetized by immersion in pasteurized tank water plus dilute benzocaine (1:2000). Filter paper fibers saturated with a gap junction-permeant dye (0.32 kDa Alexa Flour®-594 Biocytin; hereafter AFB-594) or with a mixture of a gap junction-permeant dye, 0.32 kDa AFB-594 and a gap junction-nonpermeant dye (10 kDa Dextran, Fluorescein and Biotin, Anionic, Lysine Fixable; hereafter Mini-Emerald) were surgically implanted directly into nerves innervating the deep muscles (*musculus erector analis major*, *musculus erector analis*, and *musculus depressor analis*) of the adult male gonopodium, the sexually dimorphic genitalia, and into the deep muscles of the adult female anal fin; Mosquitofish were revived and the dye was allowed to transport 6.0 h, which is sufficient to obtain Golgi-like filling of spinal motor neurons (MNs) and INs. Mosquitofish were euthanized by immersion in pasteurized tank water containing benzocaine (1:4000) and intracardially perfused with teleost buffer pH 7.4, followed with 2.0% formaldehyde made from an 8% aqueous depolymerized paraformaldehyde (PFA) diluted in teleost buffer pH 7.4. The spinal cord associated with vertebral segments 7–17 was removed, dissected-free, and post-fixed overnight with 2% PFA in teleost buffer pH 7.4. Spinal cords were covered with mounting medium (Vectashield® Vector Laboratories, Burlingame, CA) and cover-slipped. Spinal cord whole-mount preparations were viewed and digitally photographed using a Nikon CFI Super Fluor 20X objective (N.A. 0.50; W.D. 2.10) and a Nikon CFI Plan Fluor 60X 0.11–0.23 correction collar spring load objective (N.A. 0.85; W.D. 0.30) in a Nikon Eclipse 800 epi-fluorescence microscope with the appropriate single pass epi-fluorescence filters and a fluorescence illumination system (XCite™120) to attain optimal fluorescence detection efficiency. High-resolution images taken using a Qimaging Retiga Exi 12-bit CCD camera with a HRF50L1 High Resolution 0.5x coupler captured large areas and neurocytological details of MNs and interneurons (INs), specifically, commissural primary ascending interneurons (CoPA INs). The Nikon E800’s peripheral components were controlled by NIS Elements Advance Research software. Spinal cord whole-mount preparations were also viewed with a Nikon C1 Laser Scanning Confocal Microscope (LSCM) using a Nikon CFI Super Fluor 20X objective (N.A. 0.50; W.D. 2.10) and a Nikon CFI-PLAN APO 60X objective with correction collar and spring load (N.A. 0.85; W.D. 0.30). The light path consisted of 543 nm excitation, with collection using a long pass 560 nm filter or a 633 nm excitation, with collection using a long pass 650 nm filter. A PerkinElmer UltraView™ Spinning Disk Confocal scan head mounted on a Zeiss Axiovert Microscope was also used to view spinal cord whole-mount preparations.

**Figure 2:**
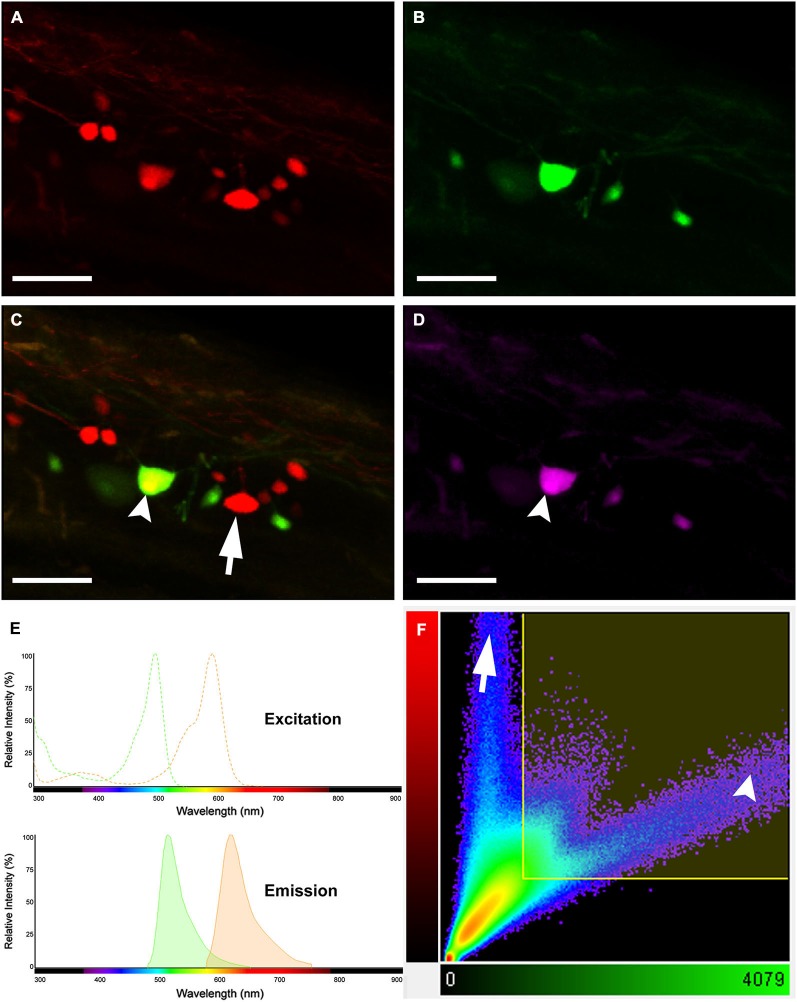
**Images showing double labeling AFB-594 (red) and Mini-Emerald (green) revealing dye-coupling between spinal motor neurons**. AFB-594 **(A)** and Mini-Emerald **(B)** retrogradely labeling revealed motor neurons from the 14th ventral root. We merge red and green channels **(C)** to demonstrate that Mini-Emerald retrograde labeling was restrict to fewer cells, presumably to motor neurons projecting to the periphery. A three-dimensional threshold co-localization analysis confirmed, as seen in the co-localization channel **(D)**, that AFB-594 retrogradely labeling diffuse to neighbor cells (arrow) in contrast to Mini-Emerald (arrow head). **(E)** Excitation and Emission spectra were carefully selected to avoid cross-talk and bleed-through of the dyes. Finally, **F** shows the co-localization scatter plot, the selected region was tresholded based on the average intensity of the somas (900) on each channel. Pearson’s coefficient in the co-localized volume for this analysis was 0.5172. Calibration bar equals 50 µm.

A simple dye-coupling assay was used to validate the gap junctional dye-coupling reported in this paper. In 100% of adult male and female Mosquitofish, retrograde labeling using either a low-molecular gap junction-permeant dye, 0.32 kDa AFB-594 (red fluorescence channel only; Figure 2A), a high-molecular weight gap junction-nonpermeant dye, 10 kDa Mini-Emerald (green fluorescence channel only; Figure 2B), or a mixture of a low-molecular gap junction-permeant dye, 0.32 kDa AFB-594 (red and green fluorescence channel overlay; Figure 2C), and a high-molecular weight gap junction-nonpermeant dye, 10 kDa Mini-Emerald (red and green fluorescence channel overlay; Figure 2C), revealed “dye-coupling” (red fluorescence in coupled spinal neurons; Figure 2C), defined as the movement of a gap junction-permeant dye (i.e., 0.32 kDa AFB-594 [red, Figure 2C]) from spinal neuron to spinal neuron.

It is important to note that the overlay channel seen in Figure 2C differs from the co-localization channel seen in Figure 2D. The overlay channel seen in Figure 2C shows all of the pixel information within a selected region of interest (ROI), including pixel information that appears in the same spatial location within the retrogradely-filled spinal neurons. The co-localization channel seen in Figure 2D shows only pixel information that appears in the same spatial location within the retrogradely-filled spinal neurons that can be seen in both the green and red fluorescence channels as seen in Figures 2B,C, respectively. We set the lower threshold limit to 900 and set the upper threshold limit based on the highest intensity level of pixels within the soma of the MN’s in a ROI. Note that this threshold was applied equally to the red and green fluorescence channels for all of the images used in our co-localization analysis. Note, too, that the excitation and emission spectra (Figure 2E) were carefully selected to avoid cross-talk and bleed-through of the gap junction-permeant and gap junction-non-permeant dyes.

The scatterplot (Figure 2F) reveals the intensity contribution of both the red fluorescence channel (vertical axis) and the green fluorescence channel (horizontal axis) in the pixel values of the analyzed images. Note that pure signal from each single channel tends to be close to the corresponding axis. In Figure 2C, the dye-coupled spinal neuron contained “pure signal” from the AFB-594 (red fluorescence channel) and is represented in the scatterplot by the pixels outside the threshold box and close to the vertical axis (see Figure 2F). In contrast, since the spinal neurons labeled with Mini-Emerald also are labeled with AFB-594, the scatterplot reveals the co-localized pixels corresponding to those spinal neurons distributed far from the horizontal axis (green fluorescence channel), thus, confirming the positive correlation between the two dyes (see Figure 2F). We used the Pearson’s correlation coefficient as a measure of the correlation of the intensity distribution between each channel. Pearson’s correlation coefficient in the co-localized volume for this analysis was 0.5172.

### Western blots

**Figure 3:**
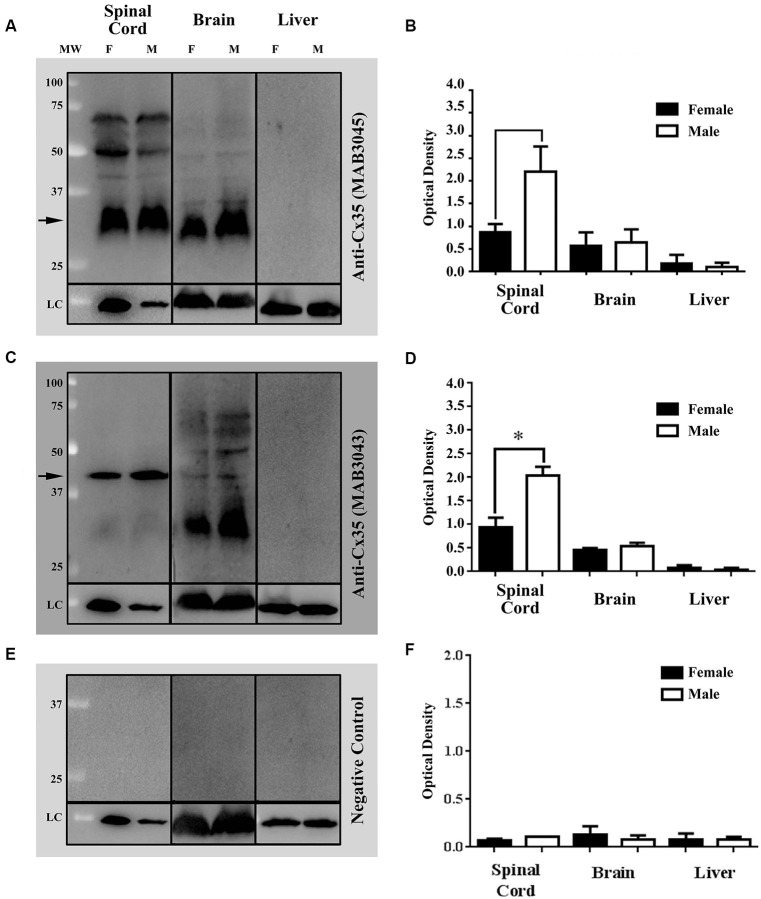
**Western blots (WB) and densitometric analysis demonstrate the specificity of two commercially available mouse anti-Cx35/36 monoclonal antibodies, MAB3045 [1:250] and (MAB3043 [1:250]) (See Figures 1A–D, respectively).** Both mouse anti- Cx35/36 monoclonal antibodies recognized single bands in both male and female spinal cords at the expected molecular weight of 35 kDa, several bands in brain tissue homogenates, and no bands (as expected) in liver tissue homogenates (see Figures 1A,C). In male and female Mosquitofish, anti-Cx35/36 monoclonal antibodies recognized single bands in spinal cord tissue homogenates, several bands in brain tissues homogenates, and no bands in liver tissue homogenates (see Figures 1A,C). WB (Figure 1C) and band density analysis (Figure 1D) of mouse anti-Cx35/36 monoclonal antibody (MAB3043 [1:250]) revealed a sex difference in Cx35/36 expression in the spinal cord tissues homogenates. In one WB (see Figure 1E), mouse anti-Cx35/36 monoclonal antibodies were omitted and the WB was incubated with only the goat anti-mouse Poly-HRP antibody. The WB and densitometric analysis (see Figures 1E,F) of this negative control validates the results of the commercially available mouse anti-Cx35/36 monoclonal antibodies by demonstrating that the specific bands recognized are not associated with a non-specific binding from the secondary antibody. OD at each protein concentration was averaged (3 blots), showing differences determined by using each potential loading control (anti-GAPDH or anti-acetylated-tubulin). Statistical differences were determined with student *t*-test analysis (* *p* < 0.05). Each lane contains 40 µg of the extracted proteins from selected tissues homogenates of adult female and male Mosquitofish. The difference in the band intensity in the loading controls (LC) of the spinal cord tissue homogenate of male and female Mosquitofish could be interpreted as an issue in the total protein loaded in the WB (see Figures 1A,C,E). However, the densitometric analysis (see Figures 1B,D,F) demonstrates a difference between males and females.

Western blots (WB) of tissue extracts from vertebrae 8–16 of intact adult Mosquitofish spinal cords, brains, and livers were used to detect female/male differences in gap junction protein expression. The spinal cords, brains, and livers of adult females (*N* = 28; groups *n* = 4) and males (*N* = 28, groups *n* = 4) were dissected out. Sample tissues were immediately frozen in dry ice and homogenized or were kept at −80°C until use. WB protocol was performed as previously described, with few minor modifications (Vega et al., 2005, 2008). Tissues were pooled, weighed, and homogenized in radioimmunoprecipitation (RIPA) buffer (1X) [Cell Signaling Technology, Beverly, MA, USA] containing 1% protease inhibitor cocktail (PIC; Sigma Aldrich, St. Louis, MO) and 1 mM phenylmethylsulfonyl fluoride (PMSF; Sigma Aldrich, St. Louis, MO). Insoluble materials were removed by centrifugation at 14,000 rpm for 5 min at 4°C. The Bradford assay (BioRad, Hercules, CA, USA) was used to estimate the approximate protein concentration of the supernatants by detecting change in absorbance using a spectrophotometer (DU730 Beckman Coulter, Brea, CA, USA). Then, samples were diluted in loading buffer (60 mM Tris–HCl [pH 6.8], 5% 2-metacaptoethanol, 2% sodium dodecyl sulfate (SDS), 10% glycerol, 0.025% Bromophenol Blue) in the appropriate volume to load 40 µg of protein per well. The supernatants, loading control, and the molecular weight marker (Precision Plus Protein Standards, Bio-Rad, Hercules, CA, USA) were electrophoresed on SDS-polyacrylamide gel (SDS-PAGE 12%). For immunoblotting, the separated proteins were transferred onto pure nitrocellulose membrane (0.45 µm, Bio-Rad, Hercules, CA, USA) for 1 hr at 4°C at a constant voltage of 100 V. Membranes were then stained with Ponceau S to corroborate transfer of proteins; then they were blocked with 5% skim milk in tris (hydroxymethyl) aminomethane [Tris] Buffered Saline with Tween® 20 (polyoxyethylene sorbitane monolaureate) [TBST] Buffer (1xTBS: 25 mM Tris Base, 150 mM NaCl, 30 mM KCl; and 0.1% Tween-20; hereafter, blocking buffer) for 1 hr. Following the blocking step, membranes were incubated overnight at 4°C with either a mouse anti-Connexin (Cx)35/36 monoclonal antibody; clone: 9D7.2 (1:250 [Cat. # MAB3043, Chemicon]; EMD Millipore, Billerica, MA, USA) or a mouse anti-Cx35/36 monoclonal antibody, clone 8F6.2 (1:250; Cat. # MAB3045, Chemicon; EMD Millipore, Billerica, MA, USA) diluted in blocking buffer. Membranes were washed several times to remove unbound Cx35/36 antibody and incubated with Goat anti-Mouse Poly-horseradish peroxidase (HRP; 1:2000; Cat. # 2230, Thermo Fisher Scientific, Suwanee, GA, USA) for 1 hr at room temperature (RT). The recognized immunoreactive bands were detected using enhanced chemiluminescence reactions according to manufacturer’s instructions (SuperSignal West Femto Maximum Sensitive Substrate [Cat.# 34095]; or SuperSignal West Dura Extended Duration Substrate [Cat. # 34075] Thermo Fisher Scientific, Suwanee, GA, USA). Then, the membranes were exposed in the ChemiDoc™ XRS+ System with Image Lab™ software (Bio-Rad, Hercules, CA, USA). For spinal cord and liver samples, membranes were stripped and reprobed for loading with anti-glyceraldehyde 3-phosphate dehydrogenase (GAPDH)-HRP conjugated (1:1000; Cat. # ab105428, Abcam, Cambridge, MA, USA). For brain samples, monoclonal anti-acetylated tubulin (1:1000; Cat. # T6793, Sigma Aldrich, St. Louis, MO, USA) was used. Then, membranes were visualized as described above. Optical density (OD) was expressed as a ratio of Cx35/36 and GAPDH or acetylated α-tubulin (Loading Control proteins [LC]) by using a densitometer and the Image Lab™ software (Bio-Rad, Hercules, CA, USA). Statistical analysis was performed with Prism 6.0 software (GraphPad Software, Inc., San Diego, CA, USA) and Office Excel 2007 (Microsoft Redmond, WA, USA). Results are reported as mean ± SEM; all the *p*-values were calculated by Student’s *t*-test (* *p* < 0.05). Western blots (Figures 3A,C) and band density (Figures 3B,D) of the mouse anti-Cx35/36 monoclonal antibodies (MAB3045 and MAB3043) show that both antibodies detect differences in Cx35/36 expression (see Figures 3A–D). Note that only mouse anti-Cx35/36 monoclonal antibody (MAB3043) revealed significant sex differences in Cx35/36 expression in spinal cord tissue homogenates (see Figures 3C,D).

### Connexin 35/36 whole-mount immunohistochemistry

We used the primary anti-Cx35/36 antibody anti-Cx35 MAB3043 (Millipore, Billerica, MA). Spinal cord whole-mounts were rinsed with phosphate buffered saline (PBS) [3 × 10 min each at RT] to remove fixative and permeabilized in a solution of PBS and 0.1% Saponin (PBSS) for 4 h at RT. Spinal cord whole-mounts were incubated with blocking buffer (3% Normal Goat Serum/PBSS) for 1 h at RT then incubated overnight at 4°C with the anti-Cx35/36 MAB3043. Following several rinses at RT with PBS [6 × 10 min each], the spinal cord whole-mounts were incubated with secondary antibody (Alexa Fluor® 488 Goat anti-Mouse [Invitrogen, Carlsbad, CA]) for 3 h at RT. Unbound antibody was removed by rinsing tissues with PBS at RT [6 × 10 min each]. The spinal cord whole-mounts were incubated with blocking buffer for 30 min at RT and were incubated with second primary antibodies or nuclear stained and mounted, as described below. All second primary antibodies were diluted in blocking buffer and incubated overnight at 4°C. Then, the spinal cord whole-mounts were rinsed several times with PBS to remove unbound second primary antibody, and the appropriate secondary antibody was added. As controls for the whole-mount immunohistochemical procedures, eye (positive control) and liver tissue (negative control) were incubated in blocking solution without primary or secondary antibody (i.e., NGS/PBSS only) followed by the standard protocol to validate the specificity of the antibody. Spinal cord, eye, and liver whole-mounts were covered with mounting medium (Vectashield® Vector Laboratories, Burlingame, CA) and cover-slipped. Spinal cord, eye, and liver whole-mounts were then viewed and digitally photographed using a Nikon CFI Super Fluor 20X objective (N.A. 0.50; W.D. 2.10) and a Nikon CFI Plan Fluor 60X 0.11–0.23 correction collar spring load objective (N.A. 0.85; W.D. 0.30) in a Nikon Eclipse 800 epi-fluorescence microscope with the appropriate single pass epi-fluorescence filters and a fluorescence illumination system (XCite™120) to attain optimal fluorescence detection efficiency. High resolution images taken using a Qimaging Retiga Exi 12-bit CCD camera with a HRF50L1 High Resolution 0.5x coupler captured large areas and neurocytological details of MNs and interneurons INs, specifically, CoPA INs.

### FRIL

The FRIL protocol has been described in detail (Rash and Yasumura, 1999; Pereda et al., 2003). The labeled spinal cord region associated with vertebral segments 7–17 (*n* = 12 females; *n* = 12 males; see labeling section above for details) was placed in a 3% low melting agarose, then transferred and embedded in 6% agarose, followed by refrigeration until fully gelled. Coronal sections (100 µm-thick) and longitudinal sections were cut using a Lancer Vibrotome 3000 (Technical Products, Inc., St. Louis, MO, USA) that maintained the tissue sections at 4°C. Spinal cord sections were infiltrated with 30% glycerol, mounted on aluminum planchettes, and frozen by contact with a liquid nitrogen-cooled metal mirror (i.e., Ultra-Freeze MF 7000; RMC Products, Tucson, AZ, USA). Frozen samples were fractured and replicated in a JEOL/RMC 9010 freeze-fracture device, then bonded to gold “index” grids by using 2.0% Lexan (GE Plastics, Pittsfield, MA, USA) dissolved in dichloroethane. After solvent evaporation at −25°C, the Lexan-stabilized samples were thawed, viewed, and digitally photographed using a 5X (0.15 N.A. Fluar) or 10X (0.5 N.A.; Plan Neofluar) objective in a Zeiss 510 Meta Laser Scanning Confocal Microscope (Carl Zeiss MicroImaging, Thornwood, NY, USA). Replicas were washed in 2.5% SDS detergent in 0.16% Tris-HCl buffer (pH 8.9) for 29 h at 48.5°C. After the initial wash in 2.5% SDS (4.0 h), the samples were digested 1.25 h in 4.0% collagenase D in 0.15 M Sorensen’s phosphate buffer (pH 7.4), followed by an additional 18–24 h in SDS solution. The replicas were rinsed in “labeling-blocking buffer” (1mg/mL LBB), then incubated for 1–1.5 hrs at 22–24°C in 1:100 dilution of anti-Cx35/36 antibody in LBB, which consists of 1.5% fish gelatin plus 10% heat-inactivated goat serum in Sorensen’s phosphate buffer (Dinchuk et al., 1987). The antibodies used for FRIL were polyclonal anti-Cx36 (36-4600) or monoclonal anti-Cx36 (39-4200 or 37-4600) from Invitrogen, polyclonal anti-Cx35 P-Ser276 from John O’Brien (Li et al., 2009), monoclonal anti-Glutamate Receptor NMDAR1 (556308, BD Biosciences), monoclonal anti-Glutamate Receptor 2 (GluR2, MAB397, Milllipore), and three anti-pannexin 1 antibodies (no specific labeling detected; therefore antibodies not separately listed). The replicas were labeled for 12–16 hrs with species-specific secondary antibodies (goat anti-rabbit or goat anti-mouse) coupled to 6-nm, 12-nm, or 18-nm gold nanoparticles (Jackson ImmunoResearch, Westgrove, PA, USA), or 30-nm gold nanoparticles (BBI). All FRIL replicas were viewed with a JEOL 2000 EX-II transmission electron microscope operated at 100 kV. Stereoscopic images (8° included angle) allowed assessment of the “sidedness” of the gold nanoparticles and the level of background immunogold labeling. Cell-specific ultrastructural markers were used to confirm cell identifications (Matsumoto et al., 1989; Rash et al., 1997), including GFAP filaments in astrocytes, spherical synaptic vesicles in axon terminals, and 10-nm E-face IMPs in glutamatergic “asymmetric” synapses *vs.* pleomorphic synaptic vesicles and 9-nm P-face IMPs in GABAergic “symmetric” synapses (Harris and Landis, 1986; DeFelipe et al., 1988; Peters et al., 1991), and labeling for NMDA and AMPA receptors in glutamatergic mixed synapses (Rash et al., 2005; also see DeFelipe et al., 1988). FRIL images were correlated with confocal microscopic images that had been obtained before SDS washing to determine specific locations of Cx35/36 gap junction puncta.

## Results

**Figure 4:**
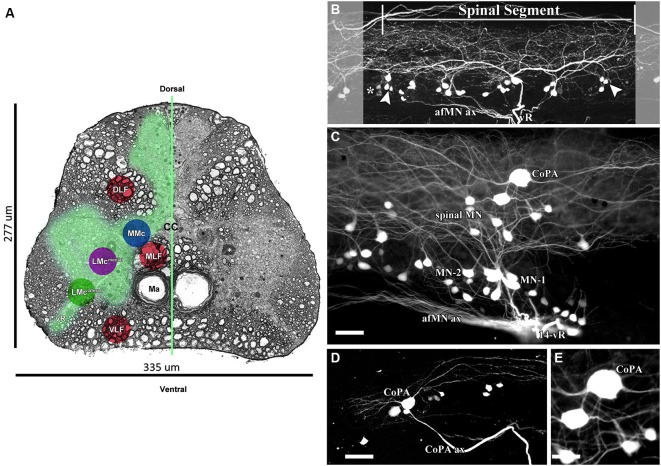
**0.32 kDa AFB-594 revealing dye-coupling between spinal motor neurons (MN) and a CoPA interneuron (IN). (A)** Serial block-face scanning electron micrograph showing a cross-sectional view of the spinal cord at the 14th ventral root. The AFB-594 motor neurons were visualized with Avidin-Biotin-Peroxidase/DAB reaction and are located in the Medial Lateral Motor Column (LMC^medial^). **(B)** Extensive dye-coupling spanned across three (14th–16th) spinal segments. **(C)** 0.32 kDa AFB-594 retrograde labeling reveal presumptive dye-coupling between MNs and an IN of the commissural primary ascending (CoPA) class. **D** and **E** higher magnification views show CoPA INs are located dorsally to the MNs in the spinal cord, have a bi-polar T-shaped dendritic arbor with fine dendrites extending rostrally and caudally from the soma into the dorsal longitudinal fasciculus, and a long axon [CoPA ax] projects ventrally. Calibration bar equals **(C and D)** 50 µm and **(E)** 30 µm.

AFB-594 retrograde labeling reveals extensive dye-coupling between MNs located in the lateral motor column (LMC) and INs located in the medial longitudinal fasciculus (MLF) of the spinal cord (Figures 4A,B). Note that this extensive dye-coupling spans three (14th–16th) spinal segments (Figure 4B), but only one spinal segment, the 14th, is shown in Figure 4B. Each spinal segment contains 44 neurons.

The 14th spinal segment, a region controlling adult male fast coital behavior, shows a more extensive and elaborate dendritic arbor (Figure 4C) than do other spinal segments. Of the 44 neurons seen in the 14th spinal segment of 12 male Mosquitofish, 28 neurons are MN-1, 7 are MN-2, and 9 are spinal MN. Of the 44 neurons seen in the 14th spinal segment in 12 female Mosquitofish, 29 were MN-1, 9 were MN-2, and 6 were spinal MN. The two phenotypes of motor neurons (MN-1 and MN-2) were identified based on the presence of basal and apical dendrites (see Figure 4C). The spinal neurons seen in Figure 4C are located within the medial motor column (MMC; Figure 4A) and the medial lateral motor column (LMC^medial^; Figure 4A). These neurons are being characterized and identified for a subsequent report. The number of MNs was assessed by counting all of the entering axons through each of the spinal segment ventral roots and correlating them with the total number of retrogradely-filled MNs. Each of the spinal segments was confirmed by the presence of “boundary neurons” (Figure 4B).

It is clear from these results that the rapid, minimally-invasive method is selective because the only spinal neurons that send axonal processes to muscles are MNs; thus, the neuronal somas and their axonal and dendritic processes extensively filled with AFB-594 are clearly distinguishable as MNs. Dye-coupling also reveals a spinal interneuron that we identify as a commissural primary ascending interneuron (CoPA IN) based on the dorso-ventral (DV) position of its soma within the MLF, and on its large slightly elongated spherical shape (see Figure 4A, and Figures 4C–E).

In keeping with the results of Hale et al. (2001), our results show that two distinct dendrites emerge from the rostral and caudal poles of the soma (Figure 4C) and run longitudinally across three spinal segments within the dorsal longitudinal fasciculus. Two dendrites (Figure 4E), together with the ventrally emerging axon, give the CoPA IN its characteristic T-shape (see Figures 4C–E). Note that dye-coupling revealed no more than one CoPA IN per spinal segment.

**Figure 5:**
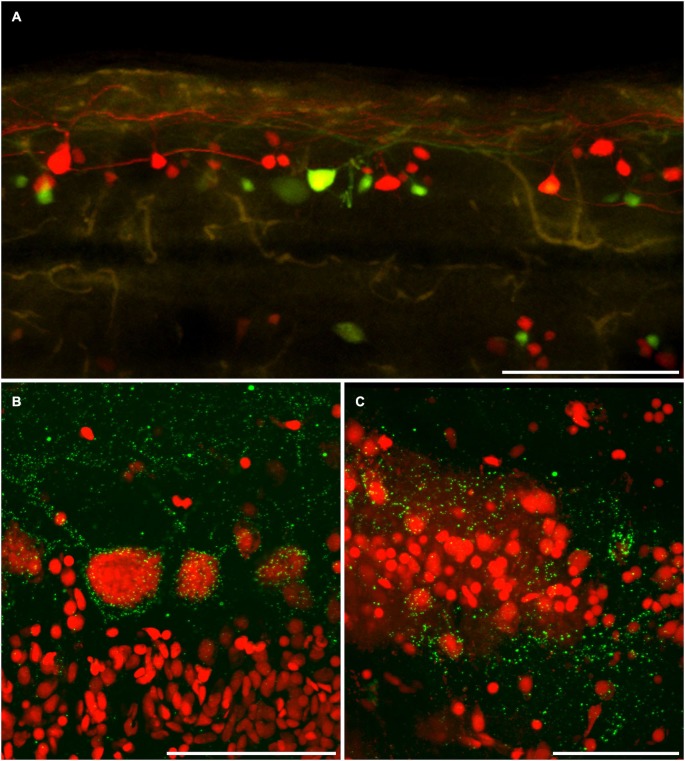
**Dye coupling is confirmed by anti-connexin 35/36 immuno-labeling. (A)** AFB-594 and Mini-Emerald retrograde labeling revealed motor neurons from the 14th ventral root, and showed the extensive labeling of presumably coupled neurons. Whole-mount immunohistochemistry with anti-Cx35 antibody confirms the widespread presence of connexins (green puncta) in both male **(B)** and female **(C)** Mosquitofish. Calibration bars equals **(A)** 200 um, **(B,C)** 50 um.

The AFB-594 dye-coupling between MNs and INs in adult female and adult male Mosquitofish as seen in Figure 5A was confirmed by performing Cx35/36 whole-mount fluorescence immunohistochemistry. Figure 5B shows the high density of Cx35/36 immuno-positive gap junction puncta covering the soma and outlining the basal, apical, and proximal dendrites of MNs in male Mosquitofish; in female Mosquitofish (see Figure 5C), the density of Cx35/36 immuno-positive gap junction puncta, especially large puncta, seems greater than it does in males.

**Figure 6:**
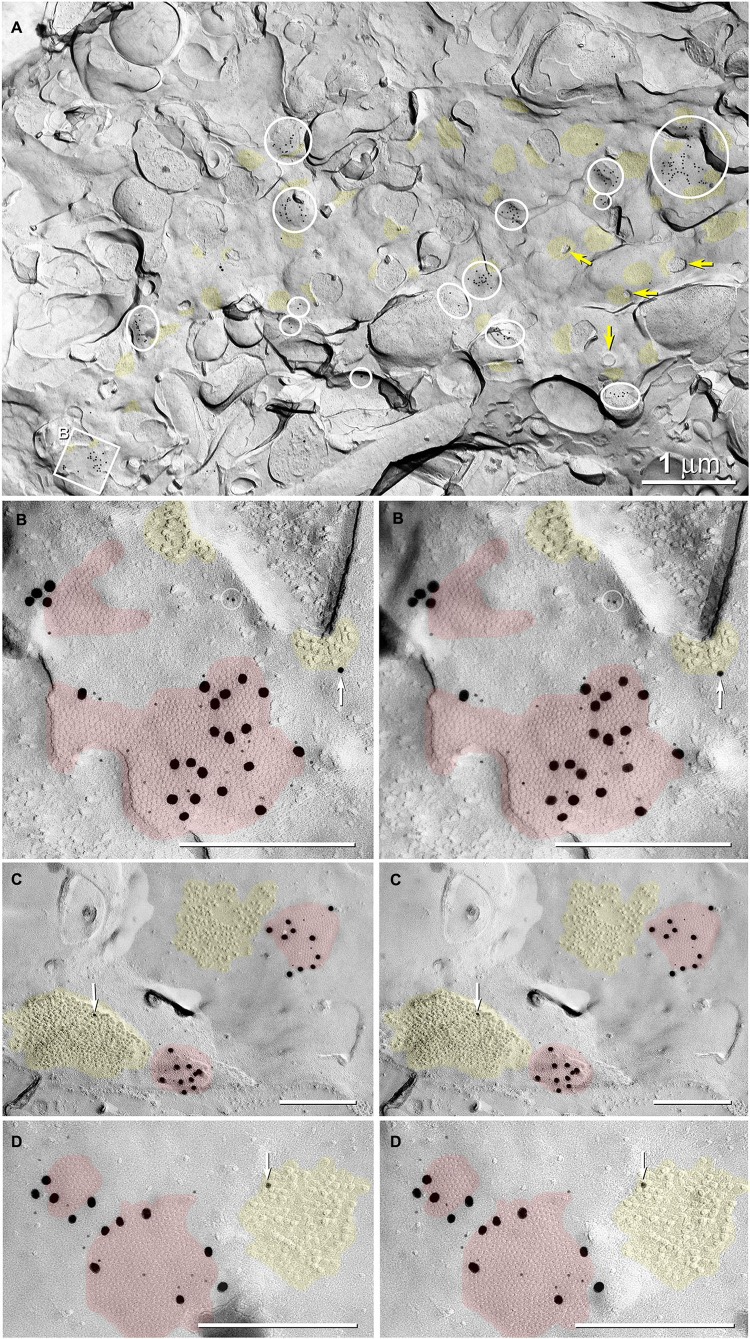
**Abundance of glutamatergic mixed synapses in adult male Mosquitofish shown at low magnification, with selected synaptic contacts shown in higher magnification stereoscopic images. (A)** The E-face of a dendrite having few spines (*yellow arrows* = cross-fractured necks of dendritic spines) is labeled extensively for Cx35 by 6-nm and 18-nm gold beads. All gap junctions are at axo-dendritic synapses (*inscribed circles* and *inscribed square B* mark 20 gap junctions in this field of view); distinctive clusters of 10-nm IMPs are weakly labeled for NMDAR1 (12- and 30-nm gold nanoparticles, *yellow overlays*; 46 PSDs in this field of view). Some cross-fractured axon terminals contain spherical synaptic vesicles. **(B)** High-magnification stereoscopic view of the inscribed square “B” in** (A)** shows two E-face gap junctions (*red overlays*) labeled for Cx35 (6-nm and 18-nm gold beads) and two postsynaptic clusters of E-face IMPs identified as glutamate receptors (*yellow overlays*) based on weak but positive labeling for NMDAR1 (12-nm gold bead,* white arrow*). Two 6-nm gold beads on top of the replica are circled to identify them as non-specific “noise”. **(C)** High-magnification stereoscopic view of two E-face gap junctions (*red overlays*) labeled for Cx35 (6-nm and 18-nm gold beads). Each gap junction has a postsynaptic cluster of IMPs (*yellow overlay*) immediately adjacent to it (i.e., within 50 nm). One IMP cluster is labeled for NMDAR1 (12-nm gold bead, *white arrow*). **(D)** High-magnification stereoscopic view of two E-face gap junctions (*red overlays*) labeled for Cx35 (6-nm and 18-nm gold beads); one postsynaptic cluster of IMPs (*yellow* *overlays*) is labeled for NMDAR1 (12-nm gold bead, *white arrow*). Unless otherwise indicated, calibration bars in all FRIL images are 0.25 µm, which corresponds to the limit of resolution of light microscopy in blue and green wavelengths.

**Figure 7:**
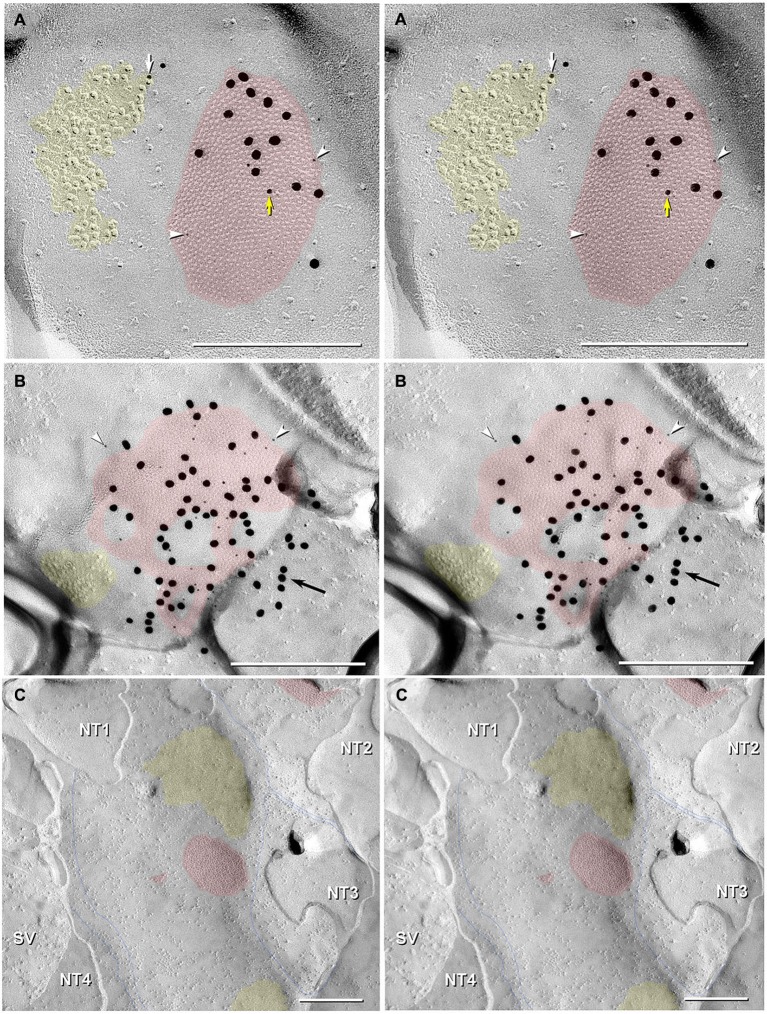
**Stereoscopic images of large plaque and reticular gap junctions in adult male Mosquitofish. (A)** Large plaque gap junction (*red overlay*) on a neuronal postsynaptic E-face labeled with antibodies against mouse/human Cx36 (36–4600, 6-nm [*white arrowheads*] and much more electron-dense 18-nm gold beads) and for GluR2 AMPA receptors (12-nm gold beads, *white arrow*) (*Yellow arrow* indicates either a 12-nm gold bead as “noise” on the Cx36-labeled gap junction or an anomalously small “18-nm” gold bead for Cx36). **(B)** High-magnification stereoscopic image of E-face of a neuronal reticular gap junction (*red overlay*) labeled for Cx35 (P-Ser276) by 6-nm (*white arrowheads*) and 18-nm gold beads. The *black arrow* points to “cryptic” labeling of connexins in an extended portion of the gap junction beneath the cross-fractured neuronal cytoplasm. Immediately adjacent to the gap junction is a cluster of E-face IMPs (*yellow overlay*, not labeled) similar to other immunogold-labeled glutamate receptor channels. **(C)** P-face image showing three of more than a dozen unlabeled postsynaptic hemiplaques (*red overlays*) in the same replica as Figures 4, 5B, where presynaptic hemiplaques are heavily labeled for Cx35 (P-Ser276). This absence of labeling in C is consistent with our demonstration in goldfish giant club ending/Mauthner cell mixed synapses that Cx35 is exclusively presynaptic and that Cx34.7 is exclusively postsynaptic (Rash et al., 2013). The postsynaptic P-face reveals distinct clusters of faintly resolvable pits (*yellow overlays*) where glutamate receptors had been removed during membrane splitting. Thin blue lines indicate probable margins of axon terminals impressed into the dendritic or somatic plasma membrane (NT1-NT4). SV = synaptic vesicles. Calibration bars are 0.25 µm.

To confirm that dye-coupling between MNs and CoPA INs was mediated by Cx35-containing-gap junctions at mixed synapses, we employed FRIL and four Cx35/36 antibodies. P-Ser276 abundantly labeled pre-synaptic hemiplaques within the neuronal gap junctions (Figures 6, 7B), as was previously reported in giant club endings on Mauthner cells (Rash et al., 2013). In 6 replicas (3 males; 3 females), FRIL reveals >115 gap junctions are immunogold labeled for Cx35/36; 97 gap junctions are found in males and 18 in females. The immunogold labeling efficiency [LE; defined as the number of gold beads *vs.* the number of connexons counted in each gap junction (Rash and Yasumura, 1999)] ranged from 1:5 to 1:50, comparable to those of previous gap junction FRIL studies (Fujimoto, 1995; Rash et al., 2001; Pereda et al., 2003).

**Figure 8:**
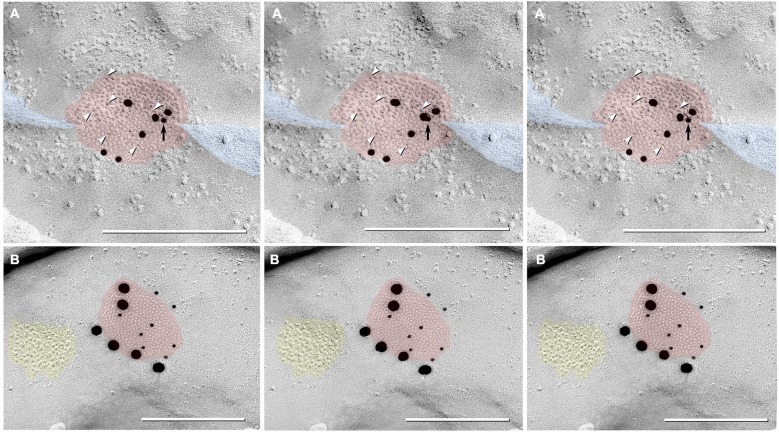
**Triplet images showing stereoscopic (left two of three images) and reverse stereoscopic images (right two images) of medium-size gap junction in adult female Mosquitofish. (A)** Classical gap junction plaque (*red overlays*) labeled by 14 6-nm gold beads (six indicated by* white arrowheads*) and six 18-nm gold beads representing Cx36 (39–4200 and 37–4600). A single 12-nm gold nanoparticle (*black arrow*), ostensibly for anti-pannexin 1 (see Section Materials and Methods), represents non-specific labeling because it is on the non-biological side (upper surface) of the replica where labeling is not possible. Note the narrowing of the extracellular space (*blue overlay*) at the point of closest membrane approach within the gap junction. **(B)** E-face image of gap junction plaque (*red overlay*) labeled for Cx36 (36–4600) by eight 12-nm gold beads and six 30-nm gold beads. Because of greatly different amino acid sequences in fish *vs.* mammalian glutamate receptors (see text), the immediately adjacent cluster of glutamate receptor-like E-face IMPs (*yellow overlay*) does not exhibit labeling for GluR2 (6-nm and 18-nm gold beads, none present). Calibration bars are 0.25 µm.

In 5 replicas (2 in adult males and 2 in adult females, each containing 6–9 spinal cord cross sections; plus 1 replica in male containing 2 longitudinal sections), we identified 35 Cx35/36 immuno-positive gap junctions in 30 appositions between neurons in the ventral horns of segment 14 in the adult Mosquitofish spinal cord (Figures 6–8); we found no gold beads on astrocyte or oligodendrocyte gap junctions (absence of labeling of glial gap junctions not shown). In one of two sagital section replicas of adult male Mosquitofish, we found 62 Cx35/36 immuno-positive gap junctions in segments containing the 8th–16th ventral roots, with >50 of those gap junctions in neurons innervating the 14th spinal segment ventral root (Figures 6–7).

Gap junctions at these glutamatergic mixed synapses are extraordinarily abundant in the 14th spinal segment (Figures 6–7), the main spinal segment that innervates the male sexually dimorphic genitalia, the gonopodium (Rosa-Molinar, 2005; Rivera-Rivera et al., 2010). Gap junctions are much less abundant in the 16th spinal segment and in the more rostral (1–7) spinal segments and in the more caudal (17–33) spinal segments of the adult (male and female) Mosquitofish spinal cord (data not shown).

Gap junctions at mixed synapses between coupled MNs and CoPA INs usually are large (>400 connexons) and are immunogold labeled by a variety of Cx35/36 antibodies. In ultrastructurally-identified mixed synapses, FRIL analysis reveals Cx35/36-labeled gap junctions adjacent to postsynaptic glutamate receptor E-face IMPs (see Pereda et al., 2003) in 43 out of 74 E-face images of mixed synapses (Figure 7,* red vs. yellow overlays*). These distinctive clusters of 10-nm E-face particles are weakly labeled for NMDA (Figure 6) or for GluR2 (Figure 7A) glutamate receptors, thereby demonstrating that both NMDA and AMPA glutamate receptors are present and intermixed in the glutamate receptor clusters. These glutamatergic mixed synapses occur primarily on dendritic shafts (Figure 6A) and are only infrequently seen on dendritic spines (not shown). The antibodies used for identifying glutamate receptors were made to mammalian amino acid sequences that vary from the sequence in fish. As a result, the glutamate-receptor antibodies are only weakly cross-reactive with fish glutamate receptors, thus yielding a low but positive level of labeling. Pereda et al. (2003) previously documented the properties of these antibodies. In any case, the number, size, and clustering of the E-face IMPs in the glutamate receptor-labeled PSDs appear identical in mammals and fish.

In addition to typical “plaques”, two large “reticular” gap junctions immunogold labeled for Cx35/36 were found in adult male Mosquitofish (Figure 7B); no reticular gap junctions have yet been found in female Mosquitofish. Reticular gap junctions are characterized by the presence of one or more oval areas that are devoid of connexon P-face IMPs / E-face pits (Kamasawa et al., 2006; Rash et al., 2007). Occasionally, the fracture plane stepped from the E-face to the P-face within the perimeter of a gap junction (Figure 8A from adult female Mosquitofish), thereby revealing the characteristic narrowing of the extracellular space (Figure 8A, blue overlay) at the contact area of gap junction coupling. Reverse stereoscopic imaging of Cx35/36 immunogold-labeled neuronal gap junctions (Figure 8; right pair of each triplet) facilitates discrimination of 6-nm gold beads from the equally electron-opaque 6–9 nm platinum-shadowed IMPs (Figure 8A).

Finally, gold beads specifically labeling Cx35 but not Cx34.7 (antibody P-Ser276; Rash et al., 2013) are restricted to presynaptic hemiplaques of neuronal gap junctions (Figures 6, 7B) and do not label nearby postsynaptic hemiplaques in the same replica (Figure 7C). We previously showed the following: two anti-Cx36 antibodies cross-react with teleost Cx35 and Cx34.7 (Mouse anti-Connexin 36 [39–4200; Figure 8A] and Ab298 [not used here]); two cross react with Cx35 but not Cx34.7 (37–4600 [Figure 8A] and Invitrogen 51–6300; not used here); two cross-react with Cx34.7 but not Cx35 (Cx34.7 IL and Cx34.7 CT; not used here); and one cross-reacts with Cx35 but has unknown cross-reactivity with Cx34.7 (36–4600 [Figures 7A, 8B]) (For antibody specificities, see Table S1 in Rash et al., 2013). In addition, phospho-specific P-Ser276 labels Cx35/Cx36 but not Cx34.7 (Figures 6, 7B,C).

Thus, the current data provide independent verification for Cx35/36 antibody specificity for labeling neuronal gap junctions but not glial gap junctions. In particular, current data confirm specificity of labeling for Cx35/Cx36 only in presynaptic hemiplaques but not in postsynaptic hemiplaques. Thus, as in goldfish, a second connexin homolog of Cx36 (likely Cx34.7; Rash et al., 2013) occurs in the otherwise unlabeled postsynaptic hemiplaques (Figure 7C). If so, these data from Mosquitofish imply that asymmetry of connexin distribution/heterotypic coupling may be widespread in teleost neuronal gap junctions.

## Discussion

The results support other studies that report gap junctions in the adult spinal cord as well as those that link gap junctions to motor behavior. Moreover, our results suggest mixed synapses have a role in the fast coital behavior of the adult male Mosquitofish, and, thus, extend prior studies (Gogan et al., 1974, 1977; Lewis, 1994; Laird, 1996; van der Want et al., 1998; Tresch and Kiehn, 2000; Kiehn and Tresch, 2002; Mentis et al., 2002; Arumugam et al., 2005; Park et al., 2011). Our findings show extensive dye-coupling between MNs and CoPA INs throughout the Mosquitofish spinal cord and permit the identification of two phenotypes of motor neurons, MN-1 and MN-2. Labeling reveals 44 neurons in the 14th spinal segment that controls the fast coital movement of the male, and it also shows that the 14th spinal segment has the most extensive and elaborate dendritic arbor. The dye-coupling study shows that the number of mixed synapses falls off precipitously in more rostral (1–13) and in more caudal (17–33) spinal cord segments in both adult male and female Mosquitofish (data not shown). The latter three findings are consistent with a fast-responding role for mixed synapses in the adult male Mosquitofish spinal cord region linked to a male-specific coital behavior.

The labeling also shows abundant anti-Cx35/36 puncta surrounding the primary basal dendrite and the soma of Mosquitofish spinal neurons. The anti-Cx35/36-labeled puncta are widely distributed throughout the Mosquitofish spinal cord. The latter finding suggests that gap junctions may link a wide constellation of spinal neurons.

Because it is well accepted that anti-Cx35/36-labeled puncta are indicative of the occurrence of gap junctions between neurons, to confirm that dye-coupling between MNs and CoPA INs was mediated by Cx35-containing-gap junctions at mixed synapses, we employed FRIL and four Cx35/36 antibodies to define the ultrastructural details of the gap junctions. Results show 62 Cx35/36 immuno-positive gap junctions in segments 8–16. In the 16th spinal segment, the more rostral (1–7) spinal segments, and the more caudal (17–33) spinal segments of the adult male and female Mosquitofish, gap junctions are not as abundant as they are in the 14th spinal segment where >50 are shown.

Thus, in the main spinal segment ventral root (14th spinal segment) that innervates the male sexually dimorphic gonopodium, gap junctions at glutamatergic mixed synapses are extraordinarily abundant, reinforcing the suggestion that they have a role in fast behavior.

Although our findings contrast with those of Matsumoto et al. (1988, 1989) who found gap junction plaques only between MNs of the spinal nucleus of the bulbocavernosus (SNB) and the dorsolateral nucleus (DLN), they are in keeping with those of others. Coleman and Sengelaub (2002) reported dye-coupling between MNs and INs associated with the rodent SNB and the DLN, both of which are sexually dimorphic motor nuclei in the lumbosacral spinal cord involved in controlling genital reflexes. Although the Coleman and Sengelaub results should be replicated in response to the Bautista and Nagy (2014) questions regarding methodological issues and the observation that dye-coupling normally occurs between neurons of the same phenotype, the dye-coupling we observed between MNs and CoPA INs in the Mosquitofish spinal cord region associated with the sexually dimorphic ano-urogenital region supports and extends the Coleman and Sengelaub (2002) results. In addition, our results are in keeping with those of Rash and coworkers who first described gap junctions at neuronal mixed synapses throughout the spinal cord of adult rat (Rash et al., 1996, 1997, 1998, 2000, 2001; Rash and Yasumura, 1999).

In short, the independent use of “dye-coupling”, whole-mount immunofluorescence for gap junction channel protein connexin 35 (Cx35), and freeze-fracture replica labeling, show the abundance and persistence of gap junctions at glutamatergic mixed synapses in adult male and female Mosquitofish and provide a means for future studies to assign specific roles to spinal neurons in the generation/regulation of a sex-specific behavior.

The results establish a base for future studies to elucidate the idea that gap junctions have a major role in regulating neuronal aborization and morphology that directly affect spinal motor activity, particularly fast motor behavior, such as the male Mosquitofish “torque/thrust” maneuver. In addition, the gap junctions found at mixed synapses between MNs and CoPA INs suggest the CoPA IN may be a novel candidate neuron for future studies of an extended role for gap junctions in coordinating fast motor behavior.

## Conflict of interest statement

The authors declare that the research was conducted in the absence of any commercial or financial relationships that could be construed as a potential conflict of interest.

## Abbreviations

AFB-594: Alexa Fluor®registered-594 Biocytin; Mini-Emerald, Dextran, Fluorescein and Biotin, 10,000 MW, Anionic, Lysine Fixable
MNs: motor neurons
CoPA IN: commissural primary ascending interneuron
CNS: central nervous system
Cx35/36: connexin 35/connexin 36
FRIL: freeze-fracture replica immunogold labeling
GFAP: glial fibrillary acid protein
IMP: intramembrane particle; LE, labeling efficiency
sCM: spinning-disk confocal microscopy
LMC^lateral^: lateral lateral motor column
LMC^medial^: medial lateral motor column
MLF: medial longitudinal fasciculus
MMC: medial motor column
DV: dorso-ventral
PBS: phosphate buffered saline
PBSS: phosphate buffered saline with saponin
RT: room temperature

## References

[B1] Arumugam H. Liu X. Colombo P. J. Corriveau R. A. Belousov A. B. (2005). NMDA receptors regulate developmental gap junction uncoupling via CREB signaling. Nat. Neurosci. 8, 1720–1726 10.1038/nn1588 16299502

[B2] Bartos M. Vida I. Frotscher M. Meyer A. Monyer H. Geiger J. R. (2002). Fast synaptic inhibition promotes synchronized gamma oscillations in hippocampal interneuron networks. Proc. Natl. Acad. Sci. U S A 99, 13222–13227 10.1073/pnas.192233099 12235359 PMC130614

[B3] Bautista W. Nagy J. I. (2014). Connexin36 in gap junctions forming electrical synapses between motoneurons in sexually dimorphic motor nuclei in spinal cord of rat and mouse. Eur. J. Neurosci. 39, 771–787 10.1111/ejn.12439 24304165 PMC3943632

[B4] Bennett M. V. (1997). Gap junctions as electrical synapses. J. Neurocytol. 26, 349–366 10.1023/A:1018560803261 9278865

[B5] Bennett M. V. (2000). Electrical synapses, a personal perspective (or history). Brain Res. Brain Res. Rev. 32, 16–28 10.1016/s0165-0173(99)00065-x 10751654

[B6] Bennett M. V. Aljure E. Nakajima Y. Pappas G. D. (1963). Electrotonic junctions between teleost spinal neurons: electrophysiology and ultrastructure. Science 141, 262–264 10.1126/science.141.3577.262 13967485

[B7] Bennett M. V. Zukin R. S. (2004). Electrical coupling and neuronal synchronization in the Mammalian brain. Neuron 41, 495–511 10.1016/s0896-6273(04)00043-1 14980200

[B8] Bonnot A. Whelan P. J. Mentis G. Z. O’donovan M. J. (2002). Spatiotemporal pattern of motoneuron activation in the rostral lumbar and the sacral segments during locomotor-like activity in the neonatal mouse spinal cord. J. Neurosci. 22:RC203 10.3410/f.1003953.42704 11826149 PMC6758517

[B9] Chen B. Liu Q. Ge Q. Xie J. Wang Z. W. (2007). UNC-1 regulates gap junctions important to locomotion in C. elegans. Curr. Biol. 17, 1334–1339 10.1016/j.cub.2007.06.060 17658257 PMC1976340

[B10] Chuang C. F. Vanhoven M. K. Fetter R. D. Verselis V. K. Bargmann C. I. (2007). An innexin-dependent cell network establishes left-right neuronal asymmetry in C. elegans. Cell 129, 787–799 10.1016/j.cell.2007.02.052 17512411

[B11] Cohen-Cory S. (2002). The developing synapse: construction and modulation of synaptic structures and circuits. Science 298, 770–776 10.1126/science.1075510 12399577

[B12] Coleman A. M. Sengelaub D. R. (2002). Patterns of dye coupling in lumbar motor nuclei of the rat. J. Comp. Neurol. 454, 34–41 10.1002/cne.10438 12410616

[B13] Connors B. W. Long M. A. (2004). Electrical synapses in the mammalian brain. Annu. Rev. Neurosci. 27, 393–418 10.1146/annurev.neuro.26.041002.131128 15217338

[B14] Costes S. V. Daelemans D. Cho E. H. Dobbin Z. Pavlakis G. Lockett S. (2004). Automatic and quantitative measurement of protein-protein colocalization in live cells. Biophys. J. 86, 3993–4003 10.1529/biophysj.103.038422 15189895 PMC1304300

[B15] DeFelipe J. Conti F. Van Eyck S. L. Manzoni T. (1988). Demonstration of glutamate-positive axon terminals forming asymmetric synapses in cat neocortex. Brain Res. 455, 162–165 10.1016/0006-8993(88)90127-8 3416182

[B16] Dermietzel R. (1998). Gap junction wiring: a ‘new’ principle in cell-to-cell communication in the nervous system? Brain Res. Brain Res. Rev. 26, 176–183 10.1016/s0165-0173(97)00031-3 9651521

[B17] Dermietzel R. Spray D. C. (1993). Gap junctions in the brain: where, what type, how many and why? Trends Neurosci. 16, 186–192 10.1016/0166-2236(93)90151-b 7685944

[B18] Dinchuk J. E. Johnson T. J. Rash J. E. (1987). Postreplication labeling of E-leaflet molecules: membrane immunoglobulins localized in sectioned, labeled replicas examined by TEM and HVEM. J. Electron Microsc. Tech. 7, 1–16 10.1002/jemt.1060070102 2464678

[B19] Domenici P. Blake R. (1997). The kinematics and performance of fish fast-start swimming. J. Exp. Biol. 200, 1165–1178 9319004 10.1242/jeb.200.8.1165

[B20] Drapeau P. Saint-Amant L. Buss R. R. Chong M. Mcdearmid J. R. Brustein E. (2002). Development of the locomotor network in zebrafish. Prog. Neurobiol. 68, 85–111 10.1016/s0301-0082(02)00075-8 12450489

[B21] Edwards D. H. Heitler W. J. Krasne F. B. (1999). Fifty years of a command neuron: the neurobiology of escape behavior in the crayfish. Trends Neurosci. 22, 153–161 10.1016/s0166-2236(98)01340-x 10203852

[B22] Fan S. Y. Ke Y. N. Zeng Y. J. Wang Y. Cheng W. L. Yang J. R. (2005). [Effects and the mechanism of carvedilol on gap junctional intercellular communication in rat myocardium]. Zhonghua Xin Xue Guan Bing Za Zhi 33, 1141–1145 16563290

[B23] Fujimoto K. (1995). Freeze-fracture replica electron microscopy combined with SDS digestion for cytochemical labeling of integral membrane proteins. Application to the immunogold labeling of intercellular junctional complexes. J. Cell Sci. 108(Pt. 11), 3443–3449 8586656 10.1242/jcs.108.11.3443

[B24] Fukuda T. Kosaka T. (2000). Gap junctions linking the dendritic network of GABAergic interneurons in the hippocampus. J. Neurosci. 20, 1519–1528 10662841 10.1523/JNEUROSCI.20-04-01519.2000 PMC6772381

[B25] Fukuda T. Kosaka T. (2003). Ultrastructural study of gap junctions between dendrites of parvalbumin-containing GABAergic neurons in various neocortical areas of the adult rat. Neuroscience 120, 5–20 10.1016/s0306-4522(03)00328-2 12849736

[B26] Fulton B. P. Miledi R. Takahashi T. (1980). Electrical synapses between motoneurons in the spinal cord of the newborn rat. Proc. R. Soc. Lond. B Biol. Sci. 208, 115–120 10.1098/rspb.1980.0045 6105652

[B27] Furshpan E. J. (1964). “Electrical transmission” at an excitatory synapse in a vertebrate brain. Science 144, 878–880 10.1126/science.144.3620.878 14149407

[B28] Furshpan E. J. Potter D. D. (1959). Transmission at the giant motor synapses of the crayfish. J. Physiol. 145, 289–325 13642302 10.1113/jphysiol.1959.sp006143 PMC1356828

[B29] Galarreta M. Hestrin S. (1999). A network of fast-spiking cells in the neocortex connected by electrical synapses. Nature 402, 72–75 10.1038/47029 10573418

[B30] Galarreta M. Hestrin S. (2001a). Electrical synapses between GABA-releasing interneurons. Nat. Rev. Neurosci. 2, 425–433 10.1038/35077566 11389476

[B31] Galarreta M. Hestrin S. (2001b). Spike transmission and synchrony detection in networks of GABAergic interneurons. Science 292, 2295–2299 10.1126/science.1061395 11423653

[B32] Gibson J. R. Beierlein M. Connors B. W. (1999). Two networks of electrically coupled inhibitory neurons in neocortex. Nature 402, 75–79 10.1038/47035 10573419

[B33] Gogan P. Gueritaud J. P. Horcholle-Bossavit G. Tyc-Dumont S. (1974). Electronic coupling between motoneurones in the abducens nucleus of the cat. Exp. Brain Res. 21, 139–154 10.1007/bf00234386 4373264

[B34] Gogan P. Gueritaud J. P. Horcholle-Bossavit G. Tyc-Dumont S. (1977). Direct excitatory interactions between spinal motoneurones of the cat. J. Physiol. 272, 755–767 592213 10.1113/jphysiol.1977.sp012071 PMC1353653

[B35] Goodenough D. A. Paul D. L. (2009). Gap junctions. Cold Spring Harb. Perspect. Biol. 1:a002576 10.1101/cshperspect.a002576 20066080 PMC2742079

[B36] Hale M. E. (2002). S- and C-start escape responses of the muskellunge (Esox masquinongy) require alternative neuromotor mechanisms. J. Exp. Biol. 205, 2005–2016 12089206 10.1242/jeb.205.14.2005

[B37] Hale M. E. Ritter D. A. Fetcho J. R. (2001). A confocal study of spinal interneurons in living larval zebrafish. J. Comp. Neurol. 437, 1–16 10.1002/cne.1266 11477593

[B38] Hamzei-Sichani F. Davidson K. G. Yasumura T. Janssen W. G. Wearne S. L. Hof P. R. (2012). Mixed electrical-chemical synapses in adult rat hippocampus are primarily glutamatergic and coupled by connexin-36. Front. Neuroanat. 6:13 10.3389/fnana.2012.00013 22615687 PMC3351785

[B39] Harper D. G. Blake R. W. (1990). Fast-start performance of rainbow-trout Salmo-Gairdneri and Northern Pike Esox-Lucius. J. Exp. Biol. 150, 321–342

[B40] Harper D. G. Blake R. W. (1991). Prey capture and the fast-start performance of Northern Pike Esox-Lucius. J. Exp. Biol. 155, 175–192

[B41] Harris K. M. Landis D. M. (1986). Membrane structure at synaptic junctions in area CA1 of the rat hippocampus. Neuroscience 19, 857–872 10.1016/0306-4522(86)90304-0 3796819

[B42] Herberholz J. Antonsen B. L. Edwards D. H. (2002). A lateral excitatory network in the escape circuit of crayfish. J. Neurosci. 22, 9078–9085 12388615 10.1523/JNEUROSCI.22-20-09078.2002 PMC6757705

[B43] Hervé J. C. Bourmeyster N. Sarrouilhe D. (2004). Diversity in protein-protein interactions of connexins: emerging roles. Biochim. Biophys. Acta 1662, 22–41 10.1016/j.bbamem.2003.10.022 15033577

[B44] Hoge G. J. Davidson K. G. Yasumura T. Castillo P. E. Rash J. E. Pereda A. E. (2011). The extent and strength of electrical coupling between inferior olivary neurons is heterogeneous. J. Neurophysiol. 105, 1089–1101 10.1152/jn.00789.2010 21177999 PMC3074410

[B45] Johnston I. I. Leeuwen J. Davies M. Beddow T. (1995). How fish power predation fast-starts. J. Exp. Biol. 198, 1851–1861 9319764 10.1242/jeb.198.9.1851

[B46] Kalb R. G. (1994). Regulation of motor neuron dendrite growth by NMDA receptor activation. Development 120, 3063–3071 7720552 10.1242/dev.120.11.3063

[B47] Kamasawa N. Furman C. S. Davidson K. G. Sampson J. A. Magnie A. R. Gebhardt B. R. (2006). Abundance and ultrastructural diversity of neuronal gap junctions in the OFF and ON sublaminae of the inner plexiform layer of rat and mouse retina. Neuroscience 142, 1093–1117 10.1016/j.neuroscience.2006.08.020 17010526 PMC1847771

[B48] Kandler K. Katz L. C. (1995). Neuronal coupling and uncoupling in the developing nervous system. Curr. Opin. Neurobiol. 5, 98–105 10.1016/0959-4388(95)80093-x 7773012

[B49] Kiehn O. Tresch M. C. (2002). Gap junctions and motor behavior. Trends Neurosci. 25, 108–115 10.1016/s0166-2236(02)02038-6 11814564

[B50] Laird D. W. (1996). The life cycle of a connexin: gap junction formation, removal and degradation. J. Bioenerg. Biomembr. 28, 311–318 10.1007/bf02110107 8844328

[B51] Lewis D. I. (1994). Dye-coupling between vagal motoneurones within the compact region of the adult rat nucleus ambiguus, in-vitro. J. Auton. Nerv. Syst. 47, 53–58 10.1016/0165-1838(94)90065-5 8188984

[B52] Lewis K. E. Eisen J. S. (2003). From cells to circuits: development of the zebrafish spinal cord. Prog. Neurobiol. 69, 419–449 10.1016/s0301-0082(03)00052-2 12880634

[B53] Li H. Chuang A. Z. O’brien J. (2009). Photoreceptor coupling is controlled by connexin 35 phosphorylation in zebrafish retina. J. Neurosci. 29, 15178–15186 10.1523/JNEUROSCI.3517-09.2009 19955370 PMC2909833

[B54] Lynn B. D. Li X. Nagy J. I. (2012). Under construction: building the macromolecular superstructure and signaling components of an electrical synapse. J. Membr. Biol. 245, 303–317 10.1007/s00232-012-9451-5 22722764 PMC3506381

[B55] Marin-Burgin A. Eisenhart F. J. Baca S. M. Kristan W. B. Jr. French K. A. (2005). Sequential development of electrical and chemical synaptic connections generates a specific behavioral circuit in the leech. J. Neurosci. 25, 2478–2489 10.1523/jneurosci.4787-04.2005 15758156 PMC6725167

[B56] Marin-Burgin A. Eisenhart F. J. Kristan W. B. Jr. French K. A. (2006). Embryonic electrical connections appear to pre-figure a behavioral circuit in the leech CNS. J. Comp. Physiol. A Neuroethol. Sens. Neural. Behav. Physiol. 192, 123–133 10.1007/s00359-005-0055-8 16205960

[B57] Marin-Burgin A. Kristan W. B. Jr. French K. A. (2008). From synapses to behavior: development of a sensory-motor circuit in the leech. Dev. Neurobiol. 68, 779–787 10.1002/dneu.20551 18383550

[B58] Matsumoto A. Arnold A. P. Micevych P. E. (1989). Gap junctions between lateral spinal motoneurons in the rat. Brain Res. 495, 362–366 10.1016/0006-8993(89)90229-1 2765937

[B59] Matsumoto A. Arnold A. P. Zampighi G. A. Micevych P. E. (1988). Androgenic regulation of gap junctions between motoneurons in the rat spinal cord. J. Neurosci. 8, 4177–4183 3183718 10.1523/JNEUROSCI.08-11-04177.1988 PMC6569466

[B60] McAllister A. K. (2007). Dynamic aspects of CNS synapse formation. Annu. Rev. Neurosci. 30, 425–450 10.1146/annurev.neuro.29.051605.112830 17417940 PMC3251656

[B61] Mentis G. Z. Diaz E. Moran L. B. Navarrete R. (2002). Increased incidence of gap junctional coupling between spinal motoneurones following transient blockade of NMDA receptors in neonatal rats. J. Physiol. 544, 757–764 10.1113/jphysiol.2002.028159 12411521 PMC2290633

[B62] Montoro R. J. Yuste R. (2004). Gap junctions in developing neocortex: a review. Brain Res. Brain Res. Rev. 47, 216–226 10.1016/j.brainresrev.2004.06.009 15572173

[B63] Muller J. F. Mascagni F. Mcdonald A. J. (2005). Coupled networks of parvalbumin-immunoreactive interneurons in the rat basolateral amygdala. J. Neurosci. 25, 7366–7376 10.1523/jneurosci.0899-05.2005 16093387 PMC6725309

[B64] Muller J. F. Mascagni F. Mcdonald A. J. (2006). Pyramidal cells of the rat basolateral amygdala: synaptology and innervation by parvalbumin-immunoreactive interneurons. J. Comp. Neurol. 494, 635–650 10.1002/cne.20832 16374802 PMC2562221

[B65] Norman K. R. Maricq A. V. (2007). Innexin function: minding the gap junction. Curr. Biol. 17, R812–R814 10.1016/j.cub.2007.07.043 17878053

[B66] Park W. M. Wang Y. Park S. Denisova J. V. Fontes J. D. Belousov A. B. (2011). Interplay of chemical neurotransmitters regulates developmental increase in electrical synapses. J. Neurosci. 31, 5909–5920 10.1523/JNEUROSCI.6787-10.2011 21508216 PMC3101639

[B67] Peinado A. Yuste R. Katz L. C. (1993a). Extensive dye coupling between rat neocortical neurons during the period of circuit formation. Neuron 10, 103–114 10.1016/0896-6273(93)90246-n 8427699

[B68] Peinado A. Yuste R. Katz L. C. (1993b). Gap junctional communication and the development of local circuits in neocortex. Cereb. Cortex 3, 488–498 10.1093/cercor/3.5.488 8260815

[B69] Pereda A. O’brien J. Nagy J. I. Bukauskas F. Davidson K. G. Kamasawa N. (2003). Connexin35 mediates electrical transmission at mixed synapses on Mauthner cells. J. Neurosci. 23, 7489–7503 12930787 10.1523/JNEUROSCI.23-20-07489.2003 PMC1805790

[B70] Pereda A. E. Curti S. Hoge G. Cachope R. Flores C. E. Rash J. E. (2013). Gap junction-mediated electrical transmission: regulatory mechanisms and plasticity. Biochim. Biophys. Acta 1828, 134–146 10.1016/j.bbamem.2012.05.026 22659675 PMC3437247

[B71] Personius K. Chang Q. Bittman K. Panzer J. Balice-Gordon R. (2001). Gap junctional communication among motor and other neurons shapes patterns of neural activity and synaptic connectivity during development. Cell Commun. Adhes. 8, 329–333 10.3109/15419060109080748 12064613

[B72] Peters A. Palay S. L. Webster H. D. (1991). The Fine Structure of the Nervous System. Neurons and Their Supporting Cells. New York: Oxford University Press

[B73] Phelan P. (2005). Innexins: members of an evolutionarily conserved family of gap-junction proteins. Biochim. Biophys. Acta 1711, 225–245 10.1016/j.bbamem.2004.10.004 15921654

[B74] Rash J. E. Curti S. Vanderpool K. G. Kamasawa N. Nannapaneni S. Palacios-Prado N. (2013). Molecular and functional asymmetry at a vertebrate electrical synapse. Neuron 79, 957–969 10.1016/j.neuron.2013.06.037 24012008 PMC4020187

[B75] Rash J. E. Davidson K. G. Kamasawa N. Yasumura T. Kamasawa M. Zhang C. (2005). Ultrastructural localization of connexins (Cx36, Cx43, Cx45), glutamate receptors and aquaporin-4 in rodent olfactory mucosa, olfactory nerve and olfactory bulb. J. Neurocytol. 34, 307–341 10.1007/s11068-005-8360-2 16841170 PMC1525003

[B76] Rash J. E. Dillman R. K. Bilhartz B. L. Duffy H. S. Whalen L. R. Yasumura T. (1996). Mixed synapses discovered and mapped throughout mammalian spinal cord. Proc. Natl. Acad. Sci. U S A 93, 4235–4239 10.1073/pnas.93.9.4235 8633047 PMC39518

[B77] Rash J. E. Duffy H. S. Dudek F. E. Bilhartz B. L. Whalen L. R. Yasumura T. (1997). Grid-mapped freeze-fracture analysis of gap junctions in gray and white matter of adult rat central nervous system, with evidence for a “panglial syncytium” that is not coupled to neurons. J. Comp. Neurol. 388, 265–292 10.1002/(sici)1096-9861(19971117)388:2<265::aid-cne6>3.0.co;2-# 9368841

[B78] Rash J. E. Olson C. O. Davidson K. G. Yasumura T. Kamasawa N. Nagy J. I. (2007). Identification of connexin36 in gap junctions between neurons in rodent locus coeruleus. Neuroscience 147, 938–956 10.1016/j.neuroscience.2007.04.061 17601673 PMC2034517

[B79] Rash J. E. Pereda A. Kamasawa N. Furman C. S. Yasumura T. Davidson K. G. (2004). High-resolution proteomic mapping in the vertebrate central nervous system: close proximity of connexin35 to NMDA glutamate receptor clusters and co-localization of connexin36 with immunoreactivity for zonula occludens protein-1 (ZO-1). J. Neurocytol. 33, 131–151 10.1023/B:NEUR.0000029653.34094.0b 15173637 PMC1892218

[B80] Rash J. E. Staines W. A. Yasumura T. Patel D. Furman C. S. Stelmack G. L. (2000). Immunogold evidence that neuronal gap junctions in adult rat brain and spinal cord contain connexin-36 but not connexin-32 or connexin-43. Proc. Natl. Acad. Sci. U S A 97, 7573–7578 10.1073/pnas.97.13.7573 10861019 PMC16587

[B81] Rash J. E. Yasumura T. Dudek F. E. (1998). Ultrastructure, histological distribution and freeze-fracture immunocytochemistry of gap junctions in rat brain and spinal cord. Cell Biol. Int. 22, 731–749 10.1006/cbir.1998.0392 10873288

[B82] Rash J. E. Yasumura T. Davidson K. G. Furman C. S. Dudek F. E. Nagy J. I. (2001). Identification of cells expressing Cx43, Cx30, Cx26, Cx32 and Cx36 in gap junctions of rat brain and spinal cord. Cell Commun. Adhes. 8, 315–320 10.3109/15419060109080745 12064610 PMC1805789

[B83] Rash J. E. Yasumura T. (1999). Direct immunogold labeling of connexins and aquaporin-4 in freeze-fracture replicas of liver, brain and spinal cord: factors limiting quantitative analysis. Cell Tissue Res. 296, 307–321 10.1007/s004410051291 10382274

[B84] Rivera-Rivera N. L. Martinez-Rivera N. Torres-Vazquez I. Serrano-Velez J. L. Lauder G. V. Rosa-Molinar E. (2010). A male poecillid’s sexually dimorphic body plan, behavior and nervous system. Integr. Comp. Biol. 50, 1081–1090 10.1093/icb/icq147 21082070 PMC2981592

[B85] Robertson J. D. Bodenheimer T. S. Stage D. E. (1963). The ultrastructure of mauthner cell synapses and nodes in goldfish brains. J. Cell Biol. 19, 159–199 10.1083/jcb.19.1.159 14069792 PMC2106865

[B86] Rosa-Molinar E. (2005). “Edwin S. Goodrich’s theory of transposition revisited: the shift to a sexually dimorphic axial formulae and nervous system in a poeciliine fish,” in First International Symposium on Livebearing Fishes, eds Uribe M. C. Grier H. J. (Homestead, Florida: New Life Publications, Inc.), 1–12

[B87] Rosa-Molinar E. Fritzsch B. Hendricks S. E. (1996). Organizational-activational concept revisited: sexual differentiation in an atherinomorph teleost. Horm. Behav. 30, 563–575 10.1006/hbeh.1996.0059 9047280

[B88] Rosa-Molinar E. Hendricks S. E. Rodriguez-Sierra J. F. Fritzsch B. (1994). Development of the anal fin appendicular support in the western mosquitofish, Gambusia affinis affinis (Baird and Girard, 1854): a reinvestigation and reinterpretation. Acta Anat. (Basel) 151, 20–35 10.1159/000147639 7879590

[B89] Rosa-Molinar E. Proskocil B. J. Hendricks S. E. Fritzsch B. (1998). A mechanism for anterior transposition of the anal fin and its appendicular support in the western mosquitofish, gambusia affinis affinis. Acta Anat. (Basel) 163, 75–91 10.1159/000046487 9873137

[B90] Rosario-Ortiz L. Rivera-Pabon S. Torres-Vazquez I. Ortiz-Perez E. G. Diaz-Perez L. Pesquera-Diaz L. (2008). The Western Mosquitofish (Gambusia affinis affinis): a new laboratory animal resource for the study of sexual dimorphism in neural circuits. Lab. Anim. (NY) 37, 263–269 10.1038/laban0608-263 18496545

[B91] Saint-Amant L. Drapeau P. (2000). Motoneuron activity patterns related to the earliest behavior of the zebrafish embryo. J. Neurosci. 20, 3964–3972 10818131 10.1523/JNEUROSCI.20-11-03964.2000 PMC6772631

[B92] Saint-Amant L. Drapeau P. (2001). Synchronization of an embryonic network of identified spinal interneurons solely by electrical coupling. Neuron 31, 1035–1046 10.1016/s0896-6273(01)00416-0 11580902

[B93] Scheiffele P. (2003). Cell-cell signaling during synapse formation in the CNS. Annu. Rev. Neurosci. 26, 485–508 10.1146/annurev.neuro.26.043002.094940 12626697

[B94] Söhl G. Maxeiner S. Willecke K. (2005). Expression and functions of neuronal gap junctions. Nat. Rev. Neurosci. 6, 191–200 10.1038/nrn1627 15738956

[B95] Sotelo C. Korn H. (1978). Morphological correlates of electrical and other interactions through low-resistance pathways between neurons of the vertebrate central nervous system. Int. Rev. Cytol. 55, 67–107 10.1016/s0074-7696(08)61887-2 389866

[B96] Spierts I. L. Leeuwen J. L. (1999). Kinematics and muscle dynamics of C- and S-starts of carp (Cyprinus carpio L.). J. Exp. Biol. 202, 393–406 9914147 10.1242/jeb.202.4.393

[B97] Sugimoto M. Sasaki S. Gotoh Y. Nakamura Y. Aoyagi Y. Kawahara T. (2013). Genetic variants related to gap junctions and hormone secretion influence conception rates in cows. Proc. Natl. Acad. Sci. U S A 110, 19495–19500 10.1073/pnas.1309307110 24218568 PMC3845116

[B98] Szabo T. M. Zoran M. J. (2007). Transient electrical coupling regulates formation of neuronal networks. Brain Res. 1129, 63–71 10.1016/j.brainres.2006.09.112 17156754 PMC1839942

[B99] Szabo T. M. Faber D. S. Zoran M. J. (2004). Transient electrical coupling delays the onset of chemical neurotransmission at developing synapses. J. Neurosci. 24, 112–120 10.1523/jneurosci.4336-03.2004 14715944 PMC6729585

[B100] Todd K. L. Kristan W. B. Jr. French K. A. (2010). Gap junction expression is required for normal chemical synapse formation. J. Neurosci. 30, 15277–15285 10.1523/jneurosci.2331-10.2010 21068332 PMC3478946

[B101] Tresch M. C. Kiehn O. (2000). Motor coordination without action potentials in the mammalian spinal cord. Nat. Neurosci. 3, 593–599 10.1038/75768 10816316

[B102] van der Want J. J. Gramsbergen A. Ijkema-Paassen J. De Weerd H. Liem R. S. (1998). Dendro-dendritic connections between motoneurons in the rat spinal cord: an electron microscopic investigation. Brain Res. 779, 342–345 10.1016/s0006-8993(97)01238-9 9473719

[B103] Vaney D. I. (1991). Many diverse types of retinal neurons show tracer coupling when injected with biocytin or neurobiotin. Neurosci. Lett. 125, 187–190 10.1016/0304-3940(91)90024-n 1715532

[B104] Vega I. E. Cui L. Propst J. A. Hutton M. L. Lee G. Yen S. H. (2005). Increase in tau tyrosine phosphorylation correlates with the formation of tau aggregates. Brain Res. Mol. Brain Res. 138, 135–144 10.1016/j.molbrainres.2005.04.015 15913839 PMC3677942

[B105] Vega I. E. Traverso E. E. Ferrer-Acosta Y. Matos E. Colon M. Gonzalez J. (2008). A novel calcium-binding protein is associated with tau proteins in tauopathy. J. Neurochem. 106, 96–106 10.1111/j.1471-4159.2008.05339.x 18346207 PMC3696493

[B106] Vervaeke K. Lorincz A. Gleeson P. Farinella M. Nusser Z. Silver R. A. (2010). Rapid desynchronization of an electrically coupled interneuron network with sparse excitatory synaptic input. Neuron 67, 435–451 10.1016/j.neuron.2010.06.028 20696381 PMC2954316

[B107] Waites C. L. Craig A. M. Garner C. C. (2005). Mechanisms of vertebrate synaptogenesis. Annu. Rev. Neurosci. 28, 251–274 10.1146/annurev.neuro.27.070203.144336 16022596

[B108] Walton K. D. Navarrete R. (1991). Postnatal changes in motoneurone electrotonic coupling studied in the in vitro rat lumbar spinal cord. J. Physiol. 433, 283–305 1668753 10.1113/jphysiol.1991.sp018426 PMC1181371

[B109] Webb P. W. (1976). The effect of size on the fast-start performance of rainbow trout Salmo cairdneri and a consideration of piscivorous predator-prey interactions. J. Exp. Biol. 65, 157–177 993700 10.1242/jeb.65.1.157

[B110] Weihs D. (1973). The mechanism of rapid starting of slender fish. Biorheology 10, 343–350 4772008 10.3233/bir-1973-10308

[B111] Whelan P. J. (2010). Shining light into the black box of spinal locomotor networks. Philos. Trans. R Soc. Lond. B Biol. Sci. 365, 2383–2395 10.1098/rstb.2009.0322 20603359 PMC2894950

[B112] Wolszon L. (1995). Cell-cell interactions define the innervation patterns of central leech neurons during development. J. Neurobiol. 27, 335–352 10.1002/neu.480270307 7673893

